# Visual three-dimensional spatial distribution of motor neurons innervating superficial limb muscles in mice

**DOI:** 10.3389/fncel.2022.904172

**Published:** 2022-07-22

**Authors:** Zhidan Qi, Shuai Han, Shen Wang, Xinyi Gu, Jin Deng, Chen Huang, Xiaofeng Yin

**Affiliations:** ^1^Department of Orthopedics and Trauma, Peking University People’s Hospital, Beijing, China; ^2^Department of Orthopaedics, Peking University Third Hospital, Beijing, China

**Keywords:** motor neuron, spinal cord, skeletal muscle, optical clearing, 3D imaging, retrograde tracing

## Abstract

The coordination of motor function in the spinal cord depends on selective connections between distinct classes of motor neurons and their target muscles. However, knowledge regarding the anatomical connections between the superficial limb skeletal muscles and the motor neurons that innervate them is limited. In this study, with a combination of the multiple retrograde tracing method with 3DISCO clearing, we explored the spatial distribution of different motor neuron pools targeting specific superficial muscles of the forelimbs or hindlimbs in mouse spinal cords, which were dominated by the radial, median, ulnar, or sciatic nerve. This study reveals the precise interrelationship among different motor neuron pools innervating limb muscles under the same space and time. The data will help to further understand the neural loop and muscular motor coordination.

## Introduction

The spinal cord is formed of various categories of neuron cells performing different biological functions. To understand the relationship between different neuron cells and muscles, we have to figure out the positional relationship of the various sorts of neuron cells, which is also one of the key problems remaining to be solved in neuroscience.

The spinal motor neurons that control limb movement are located in the lateral motor column (LMC) (Jessell, 2000). Within the LMC, motor neurons are segregated into medial and lateral compartments and project to ventral and dorsal limb muscles, respectively (Landmesser, 1978). During spinal cord development, motor neurons with common targets and afferent inputs cluster into aggregates, termed motor pools. The formation of motor pools depends not only on the early segregation of the lateral and medial subdivisions of the LMC but also on the limb-derived signals to a motor pool-specific pattern (Lin et al., 1998; Price et al., 2002). However, the relationship between the spatial distribution of motor neuron pools and corresponding specific muscles in the limb is poorly known.

The understanding of the spatial relationship of different motor neuron pools targeting specific muscles is useful not only for the diagnosis of limb dysfunction caused by motor neuron impairment but also contributes to the development of precise therapy by delivering drugs into a targeted area (Tosolini and Morris, 2016; Tosolini and Sleigh, 2017; Chen et al., 2020). Recent studies have shown that the intramuscular injections of viral vectors containing the therapeutic genes to directly target spinal cord motor neurons are a promising intervention for motor neuron diseases, such as pompe disease and amyotrophic lateral sclerosis (Elmallah et al., 2014; Chen et al., 2020; Nizzardo et al., 2020). In addition, knowledge regarding the anatomical relationship between the limb skeletal muscles and the motor neurons that supply them helps to further understand the structural basis of the neural loop, which contributes to the development of the brain-computer interface (BCI) system.

Based on the development of neural tracing techniques (Kristensson and Olsson, 1971a,b; Tosolini et al., 2013; Mohan et al., 2014, 2015), previous studies explored the distribution of motor neurons in mice innervating muscles of limbs *via* monoretrograde labeling and observed by cryosections (McKenna et al., 2000; Choi et al., 2002; Bácskai et al., 2014). And many studies showed that the motor neurons in the spinal cord were arranged in the manner of the column (Tosolini and Morris, 2012; Tosolini et al., 2013; Mohan et al., 2015). However, single-labeling tracing was employed in these studies, which could only delineate one single motor neuron pool in one specimen at the same time. And labeled neurons could only be observed *via* sectioning, which also always caused transformation and information missing due to the mechanical cutting or section holding. Recently, benefited from the great development of tissue optical clearing methods (Zhu et al., 2013; Richardson and Lichtman, 2015; Orlich and Kiefer, 2018), our group developed a multiple retrograde tracing method compatible with 3DISCO clearing, which made it possible to explore the location relationship of different motor neuron pools targeting specific muscles in mouse spinal cords under the same space and time (Han et al., 2019; Xu J.Y. et al., 2021).

In this study, with the combination of the multiple retrograde tracing method compatible with 3DISCO clearing, we explored the spatial interrelationship of different motor neuron pools targeting specific muscles of the forelimbs or hindlimbs in mouse spinal cords under the same space and time, which were dominated by the radial, median, ulnar, or sciatic nerve.

## Results

### The distribution of motor neurons dominated by the radial nerve

**FIGURE 1:**
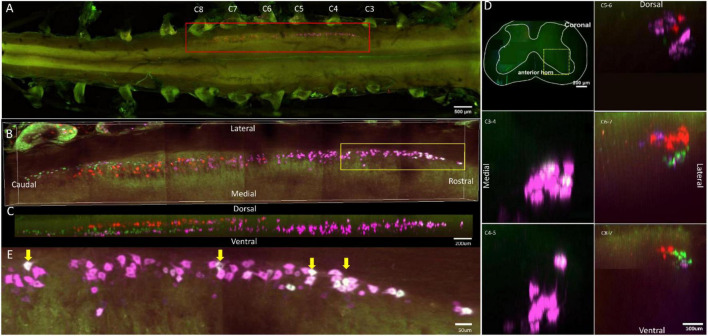
Distribution characteristics of motor neurons targeting triceps (labeled by FG, red), extensor carpi radialis longus (labeled by CTb-647, purple), and extensor carpi ulnaris (labeled by CTb-488, green). **(A)** Spatial images of multi-tracks in a holistic view. **(B,C)** The relative spatial positions of the motor neuron columns targeting different muscles in sagittal and corona viewing angles. The yellow box that outlines the region of interest was shown in panel **(E)**. **(D)** The schematic diagram of the distribution of different motor neuron columns in the anterior horn of the spinal cord. The yellow box that outlines the region of interest is amplified in the subpanels in panel **(D)**. The cross-sectional images are maximum projections of different parts of the spinal cord. The motor neurons innervating triceps (red) have little overlapping with those of extensor carpi radialis longus (purple) and extensor carpi ulnaris (green). **(E)** Motor neurons, which innervate extensor carpi radialis longus and extensor carpi ulnaris, are some double-labeled. Yellow arrow: double-labeled motor neurons. White is the overlap between purple and green colors.

**FIGURE 2:**
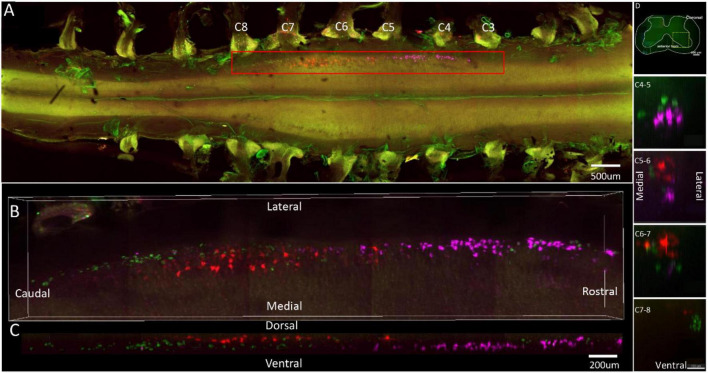
Distribution characteristics of motor neurons targeting triceps (labeled by FG, red), extensor carpi radialis brevis (labeled by CTb-647, purple), and extensor digiti minimi (labeled by CTb-488, green). **(A)** Spatial images of multi-tracks in a holistic view. **(B,C)** The relative spatial positions of the motor neuron columns targeting different muscles in sagittal and corona viewing angles. **(D)** The schematic diagram of the distribution of different motor neuron columns in the anterior horn of the spinal cord. The yellow box that outlines the region of interest is amplified in the subpanels in panel **(D)**. The cross-sectional images are maximum projections of different parts of the spinal cord. The motor neurons innervating three muscles have little overlapping.

**FIGURE 3:**
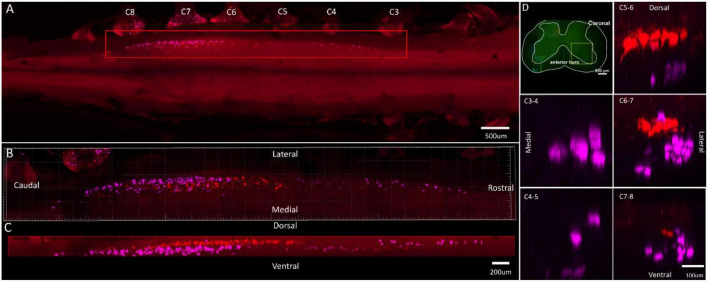
Distribution characteristics of motor neurons targeting triceps (labeled by FG, red), and extensor indicis (labeled by CTb-647, purple). **(A)** Spatial images of multi-tracks in a holistic view. **(B,C)** The relative spatial positions of the motor neuron columns targeting different muscles in sagittal and corona viewing angles. **(D)** The schematic diagram of the distribution of different motor neuron columns in the anterior horn of the spinal cord. The yellow box that outlines the region of interest is amplified in the subpanels in panel **(D)**. The cross-sectional images are maximum projections of different parts of the spinal cord. The motor neurons innervating triceps have little overlapping with extensor indicis.

**FIGURE 4:**
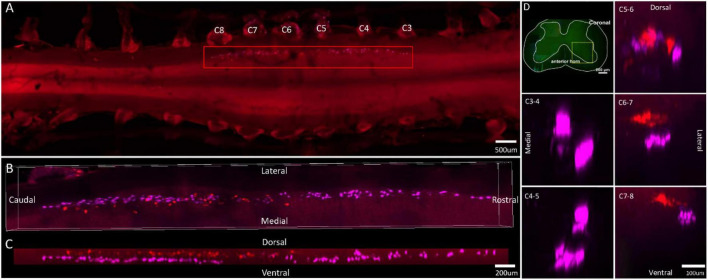
Distribution characteristics of motor neurons targeting triceps (labeled by FG, red), and extensor digitorum communis (labeled by CTb-647, purple). **(A)** Spatial images of multi-tracks in a holistic view. **(B,C)** The relative spatial positions of the motor neuron columns targeting different muscles in sagittal and corona viewing angles. **(D)** The schematic diagram of the distribution of different motor neuron columns in the anterior horn of the spinal cord. The yellow box that outlines the region of interest is amplified in the subpanels in panel **(D)**. The cross-sectional images are maximum projections of different parts of the spinal cord. The motor neurons innervating triceps have little overlapping with extensor digitorum communis.

In a holistic view, labeled neurons in the ventral horn of the spinal cord that innervated the injected muscles were distributed in the spinal segment between C3 and C8 nerve roots (Figures 1A, 2A, 3A, 4A). The spinal segment where motor neurons innervating the triceps are located was distributed between C5 and C8 nerve roots (Figures 1A, 2A, 3A, 4A). The spinal segment where motor neurons innervating the extensor digitorum communis and extensor indicis are located was between C3 and C8 nerve roots (Figures 3A, 4A). The spinal segment where motor neurons innervating the extensor digiti minimi are located was distributed between C4 and C8 nerve roots (Figure 2A). The spinal segment where motor neurons innervating the extensor carpi radialis longus and extensor carpi ulnaris are located was distributed between C3 and C8 nerve roots (Figure 1A). And the spinal segment where motor neurons innervating the extensor carpi radialis brevis are located was distributed between C4 and C7 nerve roots (Figure 2A). The individual tracers of different motor neurons above are shown in Supplementary Figures 1–4. The 3D reconstruction was successfully built on the basis of tomography images at 10 × magnification (Supplementary Videos 1–4).

In the 3D reconstruction of labeled motor neurons in the spinal cord, compared with motor neurons innervating triceps, the motor neurons, innervating the forearm extensor dominated by the radial nerve (including extensor carpi radialis longus/brevis, extensor digitorum communis, extensor digiti minimi, extensor indicis, and extensor carpi ulnaris), were located on the ventral-lateral side, and were closer to the anterior horn of gray matter in the spinal cord (Figures 1B–D, 2B–D, 3B–D, 4B–D).

An interesting phenomenon is that in the rostral part of the spinal cord, there were some double-labeled motor neurons, which innervated extensor carpi radialis longus and extensor carpi ulnaris (Figure 1E). And extensor carpi radialis longus and extensor carpi ulnaris were distant from each other but co-facilitated wrist extension. It suggested that a single motor neuron might regulate multiple functionally similar muscles.

### The distribution of motor neurons dominated by the median nerve

**FIGURE 5:**
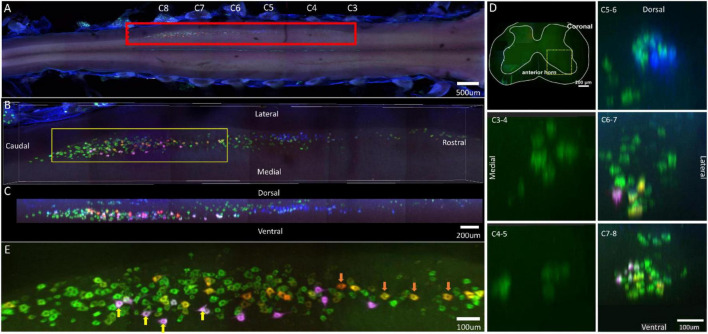
Distribution characteristics of motor neurons targeting different muscles innervated by median nerves. Motor neurons innervating flexor carpi radialis (labeled by FG, blue), palmaris longus (labeled by FR, red), flexor digitorum profundus (radialis) (labeled by CTb-647, purple), flexor digitorum superficialis (labeled by CTb-488, green). **(A)** Spatial images of multi-tracks in a holistic view. **(B,C)** The relative spatial positions of the motor neuron columns targeting different muscles in sagittal and corona viewing angles. The yellow box that outlines the region of interest is shown in panel **(E)**. **(D)** The schematic diagram of the distribution of different motor neuron columns in the anterior horn of the spinal cord. The yellow box that outlines the region of interest is amplified in the subpanels in panel **(D)**. The cross-sectional images are maximum projections of different parts of the spinal cord. **(E)** Motor neurons, which innervate flexor digitorum superficialis and flexor digitorum profundus (radialis), are some double-labeled (Yellow arrow). Motor neurons, which innervate flexor digitorum superficialis and palmaris longus, are some double-labeled (Orange arrow). Yellow is the overlap between red and green colors. White is the overlap between purple and green colors.

Labeled neurons in the ventral horn of the spinal cord that innervated the injected muscles, located in the spinal segment between the C3 and C8 nerve root. The spinal segment where motor neurons innervating the flexor digitorum superficialis are located was the longest (C3–C8 nerve root). The spinal segment where motor neurons innervating the flexor carpi radialis are located was distributed between C5 and C8 nerve roots. The spinal segment where motor neurons innervating the palmaris longus are located was distributed between C6 and C8 nerve roots. And the spinal segment where motor neurons innervating the flexor digitorum profundus (radialis) are located was distributed between C6 and C8 nerve roots (Figure 5A). The individual tracers of different motor neurons above are shown in Supplementary Figure 5. The 3D reconstruction was successfully built on the basis of the tomography images at 10 × magnification (Supplementary Video 5).

In the 3D reconstruction of labeled motor neurons in the spinal cord, the palmaris longus and flexor digitorum profundus (radialis) were distributed at the same level. Compared with flexor digitorum superficialis, palmaris longus, and flexor digitorum profundus (radialis), motor neurons innervating the flexor carpi radialis were located on the dorsal-lateral side of gray matter in the spinal cord (Figures 5B–D).

Similarly, at the caudal part of the spinal cord, there also appeared some double-labeled motor neurons, which also innervated functionally similar muscles, such as flexor digitorum superficialis/flexor digitorum profundus (radialis) and flexor digitorum superficialis/palmaris longus (Figure 5E).

### The distribution of motor neurons dominated by the ulnar nerve

**FIGURE 6:**
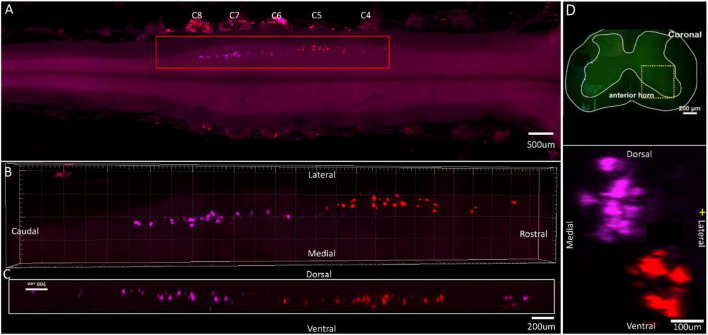
Distribution characteristics of motor neurons targeting different muscles innervated by median nerves. Motor neurons innervating flexor carpi ulnaris (labeled by FR, purple), flexor digitorum profundus (ulnaris) (labeled by CTb-647, red). **(A)** Spatial images of multi-tracks in a holistic view. **(B,C)** The relative spatial positions of the motor neuron columns targeting different muscles in sagittal and corona viewing angles. **(D)** The schematic diagram of the distribution of different motor neuron columns in the anterior horn of the spinal cord. The yellow box that outlines the region of interest is amplified in the subpanels in panel **(D)**. The cross-sectional images are maximum projections of C5–C8. The motor neurons innervating flexor carpi ulnaris have little overlapping with flexor digitorum profundus (ulnaris).

The spinal segment where motor neurons innervating the flexor carpi ulnaris are located was distributed between C6 and C8 nerve roots. The spinal segment where motor neurons innervating the flexor digitorum profundus (ulnaris) are located, was distributed between C5 and C6 nerve roots (Figure 6A). Interestingly, despite the same muscle, motor neuron pools targeting flexor digitorum profundus (radialis) were located at different segments of the spinal cord (C6–C8) (see Figure 5). The individual tracers of different motor neurons above are shown in Supplementary Figure 6. The 3D reconstruction was successfully built on the basis of tomography images at 10 × magnification (Supplementary Video 6).

In the 3D reconstruction of labeled motor neurons in the spinal cord, compared with flexor carpi ulnaris, motor neurons innervating the flexor digitorum profundus (ulnaris) were located on the ventral-lateral side and were closer to the anterior horn of gray matter in the spinal cord (Figures 6B–D).

### The distribution of motor neurons dominated by the sciatic nerve

**FIGURE 7:**
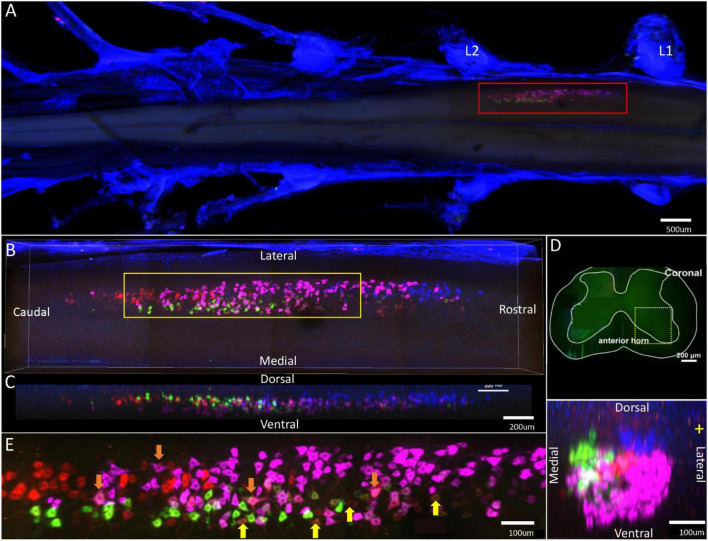
Distribution characteristics of motor neurons targeting different muscles innervated by sciatic nerves. Motor neurons innervating gastrocnemius (labeled by FR, red), tibialis anterior (labeled by FG, blue), soleus (labeled by CTb-488, green), peroneus longus/brevis (labeled by CTb-647, purple). **(A)** Spatial images of multi-tracks in a holistic view. **(B,C)** The relative spatial positions of the motor neuron columns targeting different muscles in sagittal and corona viewing angles. The yellow box that outlines the region of interest is shown in panel **(E)**. **(D)** The schematic diagram of the distribution of different motor neuron columns in the anterior horn of the spinal cord. The yellow box that outlines the region of interest is amplified in the subpanels in panel **(D)**. The cross-sectional images are maximum projections of L1–2. **(E)** Motor neurons, which innervate gastrocnemius and soleus, are some double-labeled (Yellow arrow). Motor neurons, which innervated gastrocnemius and peroneus longus/brevis, are some double-labeled (Orange arrow).

The spinal segment where motor neurons innervating the tibialis anterior, gastrocnemius, soleus, and peroneus longus/brevis are located was distributed between L1 and L2 nerve roots (Figure 7A). The individual tracers of different motor neurons above are shown in Supplementary Figure 7. The 3D reconstruction was successfully built on the basis of tomography images at 10 × magnification (Supplementary Video 7).

In the 3D reconstruction of labeled motor neurons in the spinal cord, the motor neurons innervating the peroneus longus/brevis were closer to the anterior horn of gray matter in the spinal cord. The motor neurons innervating the gastrocnemius and soleus were distributed at the same level and centrally located. The motor neurons innervating the tibialis anterior were located on the dorsal and lateral side of the anterior horn of gray matter in the spinal cord (Figures 7B–D).

In the middle part of the spinal cord, there were some double-labeled motor neurons, which innervated gastrocnemius/soleus or gastrocnemius/peroneus longus/brevis (Figure 7E).

### The distribution characteristics of motor neurons of radial, ulnar, median, and sciatic nerves

**FIGURE 8:**
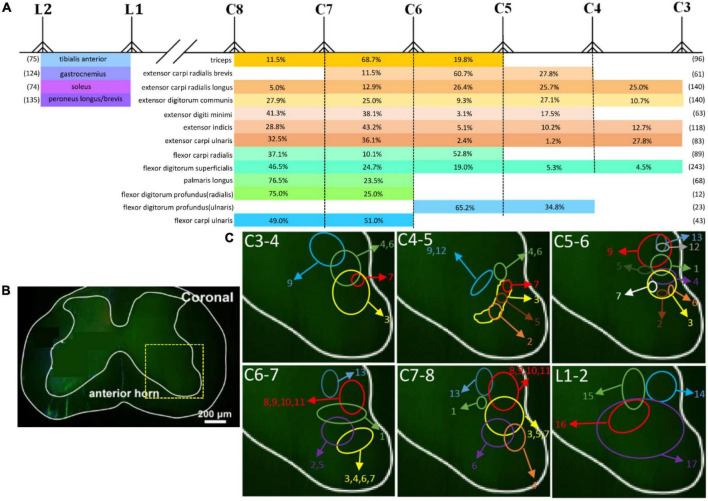
The spatial location characteristics and the number distribution of different motor neuron pools targeting skeletal muscles in spinal cord. **(A)** The number distribution of different motor neuron pools. Numbers in parentheses refer to the average total number of motor neurons innervating corresponding muscle. Percentages = number of motor neurons at single segment/total number of motor neurons innervating corresponding single muscle; *n* = 3 for each muscle. **(B)** Schematics of a coronal section of the spinal cord. The yellow box that outlines the region of interest is shown in panel **(C)**. **(C)** The distribution of different motor neuron columns in the anterior horn of the spinal cord (1: triceps; 2: extensor carpi radialis brevis; 3: extensor carpi radialis longus; 4: extensor digitorum communis; 5: extensor digiti minimi; 6: extensor indicis; 7: extensor carpi ulnaris; 8: flexor carpi radialis; 9: flexor digitorum superficialis; 10: palmaris longus; 11: flexor digitorum profundus (radialis); 12: flexor digitorum profundus (ulnaris); 13: flexor carpi ulnaris; 14: tibialis anterior; 15: soleus; 16: gastrocnemius; 17: peroneus longus/brevis).

Motor neurons in the spinal cord were arranged into relatively independent longitudinal columns. The distribution was shown by dividing the spinal segments where the labeling neurons were distributed. Then we counted the average total number of motor neurons innervating corresponding muscle and the average percentage at a single segment (shown in Figure 8A). We further reconstructed the motor neurons’ distribution and showed relative location characteristics of motor neurons in the spinal cord by measuring the relative distance of each motor neuron pool to the edge of the spinal cord (Figure 8B). The spatial relationship of different motor neuron pools in one spinal cord is shown in Figure 8C.

## Discussion

Motor neuron pools are an organizational scheme in the spinal cord that cluster functionally related neuronal soma as spatially distinct groups (Romanes, 1964). However, little is known of the mutual spatial distribution of different functional motor neuron pools. In this study, with the combination of the multiple retrograde tracing method compatible with 3DISCO clearing, we produce a detailed map of the spatial distribution of different motor neuron pools targeting specific muscles of four limbs in mouse spinal cords, which are dominated by the radial, median, ulnar, or sciatic nerve. And we preliminarily explore the mutual position of different functional motor neuron pools.

Based on the results above, we observe that the motor neuron pools targeting different muscles of four limbs are usually regularly distributed:

1. The motor neuron pools targeting the synergistic or antagonistic muscles are distributed in the closer spinal cord segments. For example, the motor neuron pools targeting the extensor carpi ulnaris and flexor carpi ulnaris, which are antagonist muscle groups, are both mainly distributed between C6 and C8. The motor neuron pools targeting the extensor carpi radialis brevis and extensor carpi radialis longus, which are synergist muscle groups, are both mainly distributed between C4 and C6. The motor neuron pools targeting the flexor digitorum superficialis and extensor digitorum communis, which innervate 2–5 fingers and are antagonist muscle groups, are both distributed between C3 and C8. Thus, the spatial contact in the similar spinal cord segments of motor neuron pools is the structural basis of mutual signal delivery, which results in different muscular functions.
2. Different motor neuron pools in the same segment display differences in spatial distribution. The motoneuron columns that innervate distal muscles are closer to the edge of the anterior horn of gray matter in the spinal cord. For example, the motoneuron columns targeting muscles of the wrist or finger are located more laterally and ventrally than the targeting triceps. The differences in the spatial distribution of motor neuron pools might result from different development sequences. Shortly after birth, motor neurons that settle in the median motor column project their axons to the axial and body wall muscles, whereas motor neurons in the lateral motor column (LMC) project axons selectively to limb muscles (Hollyday, 1980; Jessell, 2000). At the same time, the motoneuron columns that innervate flexor muscles are located more medially in the same spinal cord segment than the motoneuron columns that innervate extensor muscles. For example, the motoneuron columns targeting flexor carpi ulnaris are located more medially than that targeting extensor carpi ulnaris and the motoneuron columns targeting gastrocnemius are located more medially than those targeting tibialis anterior. However, the underlying cause of such spatial distribution of specific sets of neurons needs further exploration.
  The spatial location of different motor pools might represent different muscle functions by influencing the pattern of motor neuron inputs from local circuit interneurons, descending supraspinal neurons, or primary afferent fibers. The spatial location of motor neurons is strongly associated with corresponding muscle function. Once the spatial location changes, the axonal projections in the peripheral changes accordingly (Eisen, 1991). The accurate spatial localization of motor neuron cell bodies helps to speculate corresponding muscle function.
3. An interesting phenomenon is that part of motor neurons is double-labeled. It suggests that a single motor neuron might regulate multiple functionally similar muscles. For example, the flexor digitorum superficialis and flexor digitorum profundus co-facilitate finger flexion, and some motor neurons innervating both muscles are also double-labeled. Likewise, flexor digitorum superficialis and palmaris longus co-facilitate wrist flexion; gastrocnemius and soleus co-facilitate plantars flexor. In addition, extensor carpi radialis longus and extensor carpi ulnaris, which are distant from each other but co-facilitate wrist extension, and motor neurons innervating both muscles are also double-labeled. To exclude this possibility that leakage of tracer results in double-label, when dissecting the median nerve or ulnar nerve separately, motor neuron pools targeting flexor digitorum profundus are located separately and not double-labeled. The results above suggest that it is easier to double-label motor neuron pools targeting muscles with a similar function, but not a similar location. Similarly, the “one-to-many” interaction pattern is also found in the M1 neurons in the brain cortex, which make direct connections to the multiple motoneuron pools in the spinal cord (Cheney and Fetz, 1985; Jackson et al., 2003). But some questions still require to be further investigated: Is there any difference between double-labeled neurons and motor neurons in the traditional sense? Does the excitation of double-labeled neurons lead to inactivation of two muscles or selective excitation of just one muscle?
4. If the muscle is dominated by different nerves, the motor neuron pools targeting the different parts of the muscle are distributed separately, not mixed up. The flexor digitorum profundus is innervated by the median nerve and ulnar nerve together. When dissecting the median nerve or ulnar nerve separately, motor neuron pools targeting flexor digitorum profundus are located at different segments of the spinal cord. It suggests that when the same muscle takes on different functions, such as flexion of different fingers, it is innervated by different motoneurons to present a more refined movement. It also suggests different segments of motor neuron pools discriminate different functions.

The study still contains some limitations: (1) To reveal deep muscles, we have to open shallow muscles, which are easily accessible to damage the innervation of deep muscles and thus result in failure of retrograde tracing. So this study mainly focuses on shallow muscles and does not obtain neuron distribution regularity of all muscles. (2) Although the spatial organizations of the motor neurons could be preserved after clearing (Ertürk et al., 2011; Launay et al., 2015; Žygelytë et al., 2016), the dehydration procedure of clearing methods causes the size decrease due to tetrahydrofuran (Pan et al., 2016). Thus, we could only obtain the relative position relationships of multiple motor neuron pools, and accurate quantitation requires transformation according to the proportion (Becker et al., 2012; Ertürk et al., 2014). (3) One drawback of the use of retrograde tracers is that they give only an approximation of the total number of motor neurons targeting specific muscles. Indeed, although great care is taken to minimize inter-injection variability, the variability in the number of labeled neurons between injections in the same muscle is related to a large extent to differences in intake and/or transport of retrograde tracers. In these instances, the number of labeled motor neurons can actually be considered an underestimation of the actual population (Nicolopoulos-Stournaras and Iles, 1983; Tosolini and Morris, 2012; Xu J. et al., 2021). (4) A previous study demonstrates that the tibialis anterior is between L2 and L4 and gastrocnemius is between L3 and 4 (Mohan et al., 2014). On the one hand, spinal segments are carefully identified by counting the sequence of vertebrae not by dissecting the dorsal roots of the spinal nerves to their point of origin in the spinal cord in our study. On the other hand, it might be a result of the tracers not targeting the entire motor endplate region, as demonstrated in previous studies (Tosolini et al., 2013; Mohan et al., 2014; Tosolini and Morris, 2016).

There is still a lack of effective pharmacological treatments for many motor neuron diseases, including spinal muscular atrophy (SMA). In recent studies, intramuscular injection is considered the most specific and ideal approach for gene therapy in lower motor neuron diseases (Boulis et al., 2003; Towne et al., 2010). Knowledge of the neuroanatomical tract between motor neurons and skeletal muscles is the basis of intramuscular injection. In this study, in combination with tissue clearing, retrograde tracing, and whole-mount analysis, the mutual spatial distribution of different functional motor neuron pools in the same spinal cord and the anatomical relationship between the skeletal muscles and the motor neurons, will be updated and refined. It might have significant implications for the development of therapies in mouse models of limb dysfunction including models of motor neuron diseases, and for studies aiming to restore motor function in a specific muscle or muscle groups.

In this study, we inject the retrograde tracers into muscles to explore the spatial distribution of different motor neuron pools targeting specific muscles of four limbs in mouse spinal cords. The method is designed from the effector view to analyze the spatial inter-relationship of motor neurons targeting the different functional muscles in the background of overall imaging. The distribution of spinal motor neuron pools in normal mice could provide the structural basis for the studies on the link between different functional motor neurons.

## Materials and methods

### Anatomy of the muscles and their innervation

The muscles are divided into four groups according to different innervation, which are injected with retrograde tracers (see Table 1). The morphology of the different muscles is shown in Supplementary Figure 8.

**TABLE 1:** Muscles that are injected with retrograde tracers and the corresponding nerves (Greene, 1935).

Nerve	Muscle
Radial nerve	Triceps, extensor carpi radialis longus, extensor carpi radialis brevis, extensor digitorum communis, extensor digiti minimi, extensor indicis, extensor carpi ulnaris
Ulnar nerve	Flexor carpi ulnaris, flexor digitorum profundus (ulnaris) (median nerve dissection)*
Median nerve	Flexor carpi radialis, flexor digitorum superficialis, palmaris longus, flexor digitorum profundus (radialis) (ulnar nerve dissection)*
Sciatic nerve	Tibialis anterior, gastrocnemius, soleus, peroneus longus/brevis

*The flexor digitorum profundus is innervated by the median nerve and ulnar nerve together. When injecting muscles innervated by the median nerve, we dissect the ulnar nerve to explore the distribution of motor neurons innervating flexor digitorum profundus (radialis) and vice versa.

### Animal care

All animal treatments and procedures of this study were performed in strict accordance with recommendations in the Institutional Animal Care Guidelines and approved ethically by the Administration Committee of Experimental Animals of Peking University People’s Hospital (Permit Number: 2020PHE089). To assure three repetitions per group, 21 adult female SPF-grade C57BL/6 strain mice, 6–8 weeks old, weighing 20–25 g, were purchased from Beijing Vital River Laboratory Animal Technology Co. Ltd. (Beijing, China). The animals were maintained on a 12/12 h light-dark cycle with *ad libitum* access to food and water. All efforts were made to minimize suffering.

### Tracer preparation

Four retrograde tracers were used for labeling the motor neurons in the spinal cord as follows: FG (Fluorochrome, Denver, CO, United States) was dissolved at 5% in distilled water and saved at 4°C. Fluoro-Ruby (FR, Molecular Probes, Eugene, OR, United States) and CTb (Recombinant), Alexa Fluor TM 488, and 647 Conjugate (CTb-488, CTb-647, Molecular Probes, Eugene, OR, United States) were dissolved at 10%, respectively, in normal saline and 1X phosphate-buffered saline (PBS) and saved at –20°C. All these tracers were kept on the ice during preparation and injection.

### Surgical procedure

The mice were anesthetized by intramuscular injection of keta-mine-xylazine (100 mg/kg ketamine; 10 mg/kg xylazine) and maintained under anesthesia with isoflurane/oxygen. An incision was made to reveal the muscles. Fascia was minimally disrupted over the muscles of interest and no blood vessels were disrupted. The muscles were divided into four groups according to different innervation, which were injected with retrograde tracers (see Table 1 above). When injecting the deep muscles, such as soleus, we incised the muscular space between the gastrocnemius and lateral muscles, lifted the gastrocnemius, and then the dark red soleus muscle was exposed. The schematic diagram is shown in our previous study (Han et al., 2019).

### Tracing procedure

**FIGURE 9:**
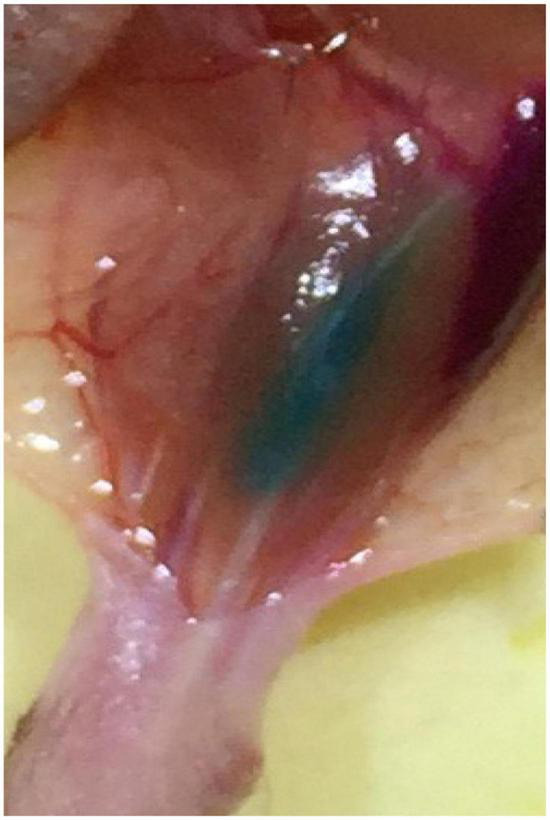
The image with the tracer injected into the muscle.

Multiple injections were slowly performed by use of a 5 ul Hamilton syringe under microscopic guidance. To improve the labeling efficiency, the tracers were injected until the muscles were completely colored, ensuring that the whole muscles were filled with the tracers (see Figure 9). Depending on the size of the muscles, the injection volume was approximately 1–5 ul (see Table 2).

**TABLE 2:** The retrograde tracers and injection volume of muscles.

Muscle	Tracer	Injection volume
Tibialis anterior	FG	2 ul
Gastrocnemius	FR	4 ul
Soleus	CTb-488	1 ul
Peroneus longus/brevis	CTb-647	2 ul
Triceps	FG	5 ul
Extensor carpi ulnaris	CTb-488	1 ul
Extensor carpi radialis longus	CTb-647	1 ul
Extensor carpi radialis brevis	CTb-647	1 ul
Extensor digiti minimi	CTb-488	2 ul
Extensor indicis	CTb-647	1 ul
Extensor digitorum communis	CTb-647	2 ul
Flexor carpi radialis	FG	1 ul
Palmaris longus	FR	1 ul
Flexor digitorum profundus(radialis)	CTb-647	1 ul
Flexor digitorum superficialis	CTb-488	2 ul
Flexor carpi ulnaris	FR	2 ul
Flexor digitorum profundus(ulnaris)	CTb-647	1 ul

### Spinal cord harvest

Based on several previous papers which showed that 1 week was enough to provide adequate cell labeling (Hirakawa et al., 1992; Novikova et al., 1997; Choi et al., 2002), we chose 1 week here to allow sufficient retrograde transport of the tracers. After the intramuscular injections, the mice were kept for 7 days to allow for optimal retrograde transport of neuronal tracers. Then, the mice were deeply anesthetized and perfused transcardially with 37°C saline followed by 4°C 4% paraformaldehyde (PFA; Sigma-Aldrich, St. Louis, MO, United States dissolved by 1X PBS). After transcardial perfusion, the intact spinal cord was removed between C1 and T2 region or between T12 and S2 region to ensure the entire motor neuron pool was covered. Spinal nerve roots were left to recognize the specific segments of the spinal cord. Then, the spinal cord was fixed with the post-fixation solution (4% PFA) overnight. The sample was immobilized with microsutures on a piece of flat-folded aluminum foil during the fixation and optical clearing procedure to prevent deformation.

### Optical clearing

**FIGURE 10:**
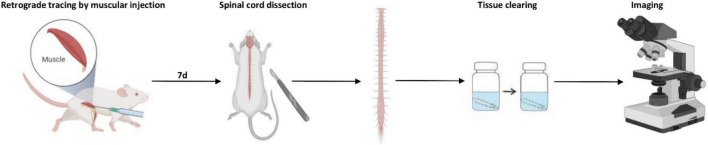
The schematic process of 3D imaging for the spinal cord.

The optical clearing procedure was performed as the reported 3DISCO method (Ertürk et al., 2012) and our previous study (Han et al., 2019). The entire experimental process of 3D imaging for the spinal cord is shown in Figure 10.

### Imaging

The spinal cord was mounted in 100% DBE with two pieces of cover glass. The confocal microscope (Zeiss LSM 780) was set at a bit depth of 8, averaging of 2, laser intensity of 4–15, pin-hole of 1.0 airy units, and gain of 650. Imaging was performed at 5X magnification for the overall view of the spinal cord and nerve roots followed by 10X magnification for a positive signal zone. Best-signal mode of Zeiss LSM 780 was chosen to scan, and the filters were automatically optimized. Adjustments were made to the gain to obtain a brighter image and maximum intensity projection (MIP) was performed to display the distribution of the signals. Three-dimension (3D) reconstruction was performed based on scanning images of 10X magnification to build the stereoscopic distribution of the labeled neurons. Imaris 7.6.0 was used respectively for the MIP and 3D reconstruction.

## Data availability statement

The original contributions presented in the study are included in the article/Supplementary Material, further inquiries can be directed to the corresponding author.

## Ethics statement

The animal study was reviewed and approved by the Administration Committee of Experimental Animals of Peking University People’s Hospital.

## Author contributions

ZQ performed most of the experiments on the mice with the help of SH. SW performed and analyzed the three-dimensional reconstruction of motor neurons. XG analyzed the three-dimensional reconstruction. JD performed the statistical analysis. CH wrote the manuscript. XY initiated, conceived, and supervised the project. All authors contributed to the experimental design and interpretation, commented on the manuscript, and approved the submitted version.

## Conflict of interest

The authors declare that the research was conducted in the absence of any commercial or financial relationships that could be construed as a potential conflict of interest.

## Publisher’s note

All claims expressed in this article are solely those of the authors and do not necessarily represent those of their affiliated organizations, or those of the publisher, the editors and the reviewers. Any product that may be evaluated in this article, or claim that may be made by its manufacturer, is not guaranteed or endorsed by the publisher.
