# Supplementary material for: Visual three-dimensional spatial distribution of motor neurons innervating superficial limb muscles in mice
Source: Front Cell Neurosci. 2022 Jul 22;16:904172. doi: 10.3389/fncel.2022.904172 (PMC9354668; doi:10.3389/fncel.2022.904172)
Supplement: Supplementary file 1 [file Data_Sheet_1.PDF]

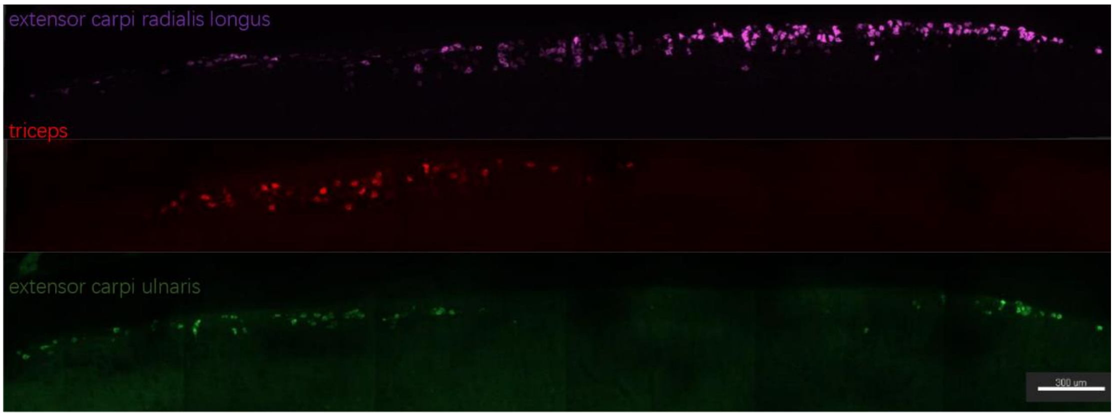

Supplementary Figure 1 Distribution characteristics of motor neurons targeting triceps (labelled by FG, red), extensor carpi radialis longus (labelled by CTb-647, purple) and extensor carpi ulnaris (labelled by CTb-488, green).

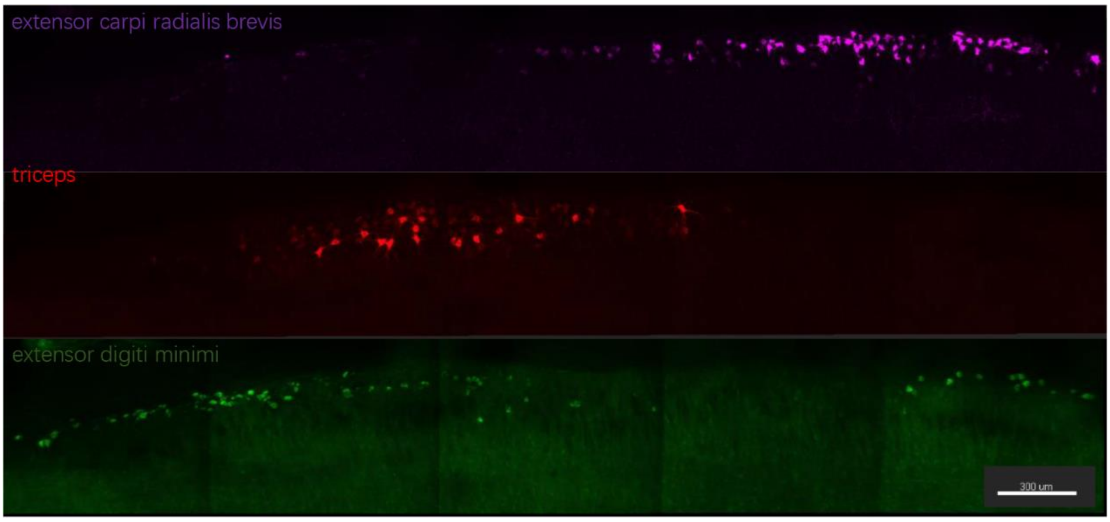

Supplementary Figure 2 Distribution characteristics of motor neurons targeting triceps (labelled by FG, red), extensor carpi radialis brevis (labelled by CTb-647, purple), and extensor digiti minimi (labelled by CTb-488, green).

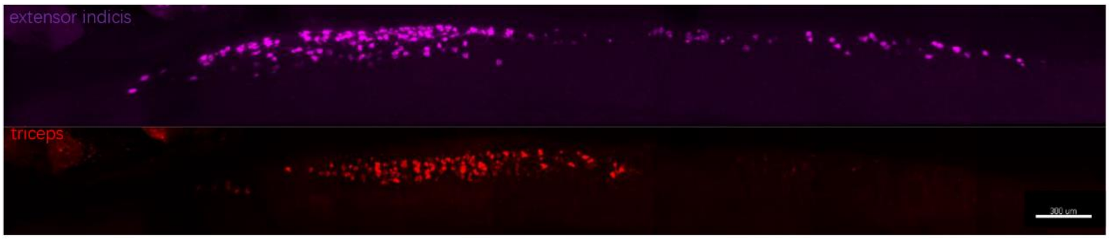

Supplementary Figure 3 Distribution characteristics of motor neurons targeting triceps (labelled by FG, red), and extensor indicis (labelled by CTb-647, purple).

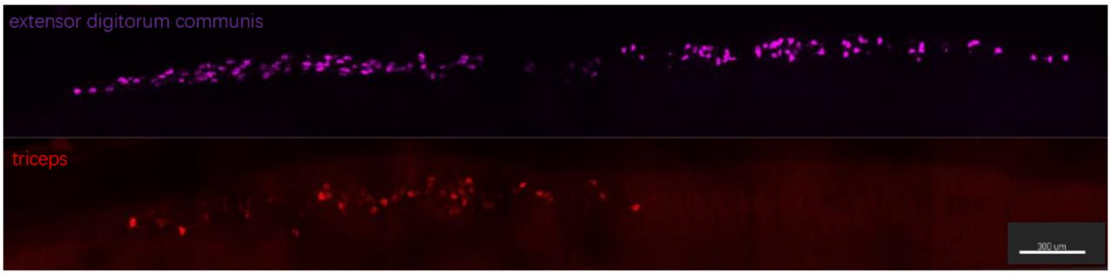

Supplementary Figure 4 Distribution characteristics of motor neurons targeting triceps (labelled by FG, red), and extensor digitorum communis (labelled by CTb-647, purple).

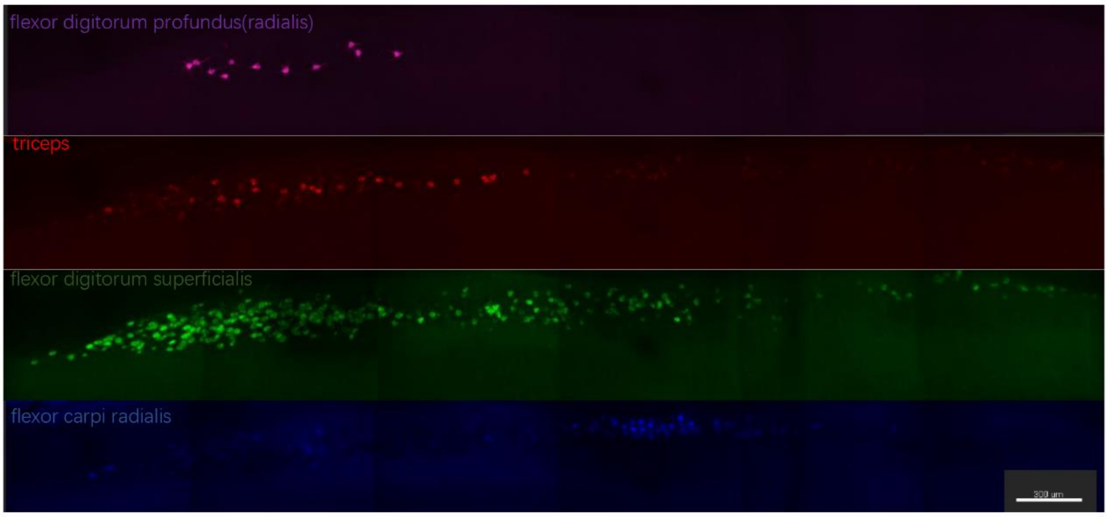

Supplementary Figure 5 Distribution characteristics of motor neurons targeting flexor carpi radialis (labelled by FG, blue), palmaris longus (labelled by FR, red), flexor digitorum profundus(radialis) (labelled by CTb-647, purple), and flexor digitorum superficialis (labelled by CTb-488, green).

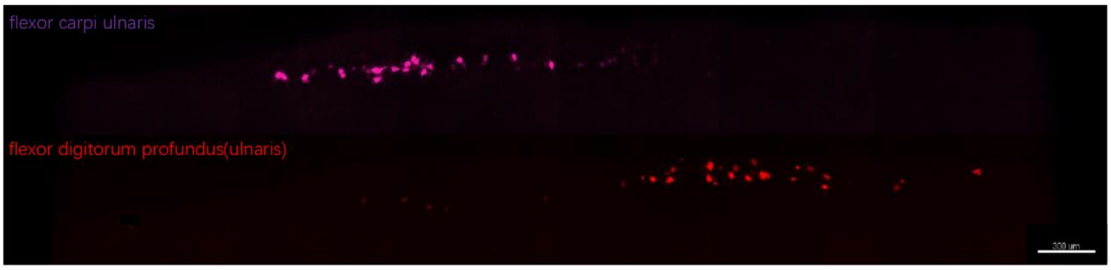

Supplementary Figure 6 Distribution characteristics of motor neurons targeting flexor carpi ulnaris (labelled by FR, purple), and flexor digitorum profundus(ulnaris) (labelled by CTb-647, red).

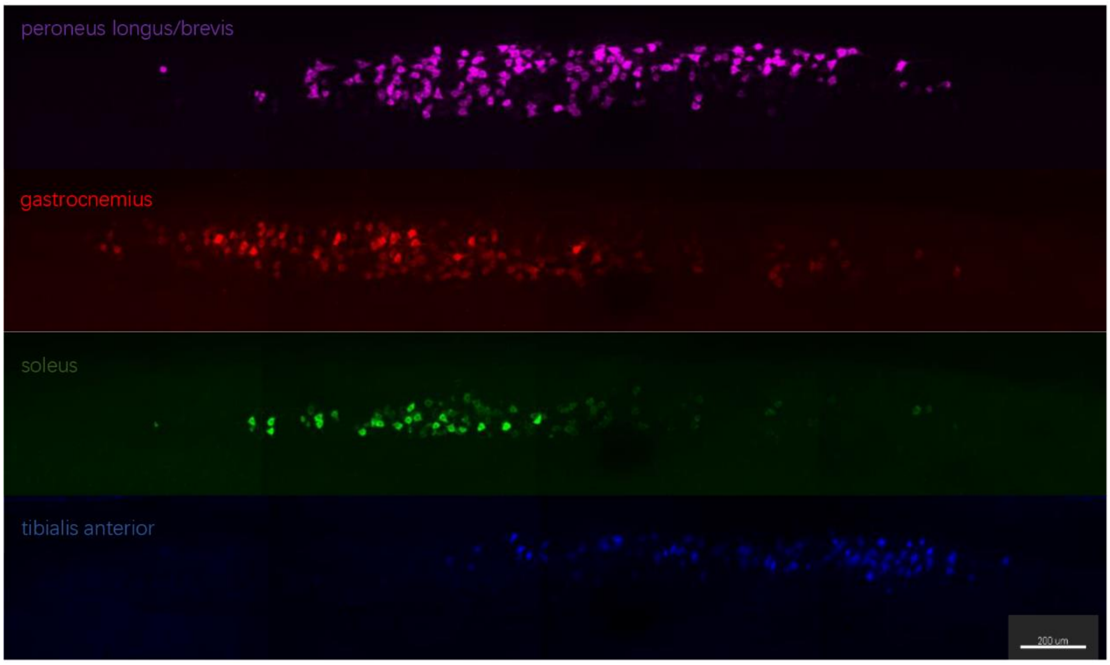

Supplementary Figure 7 Distribution characteristics of motor neurons targeting gastrocnemius (labelled by FR, red), tibialis anterior (labelled by FG, blue), soleus (labelled by CTb-488, green), and peroneus longus/brevis (labelled by CTb-647, purple).

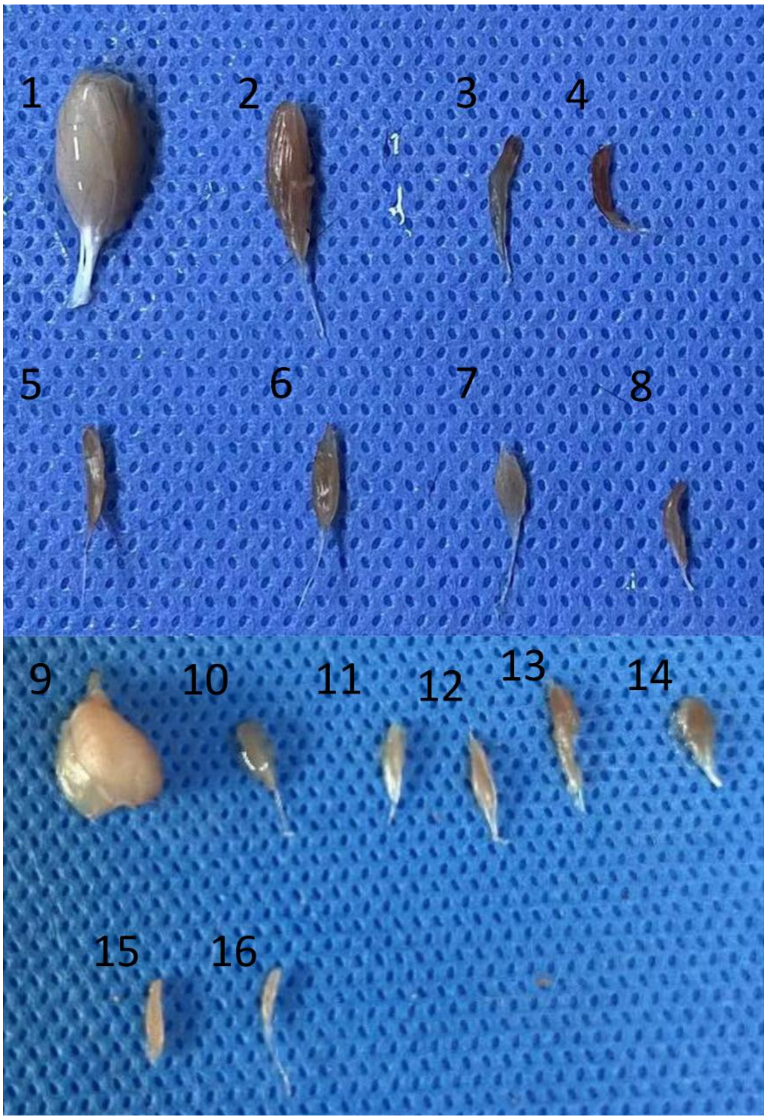

Supplementary Figure 8 Morphology of the different muscles. 1: gastrocnemius;2: tibialis anterior;3: peroneus longus/brevis;4: soleus;5: extensor carpi radialis brevis;6: extensor carpi radialis longus;7: extensor digitorum communis;8: flexor carpi radialis;9:triceps;10:extensor carpi ulnaris;11:palmaris longus;12: flexor carpi ulnaris;13: flexor digitorum superficialis;14: flexor digitorum profundus;15: extensor digiti minimi;16: extensor indicis.
